# Color Regeneration from Reflective Color Sensor Using an Artificial Intelligent Technique

**DOI:** 10.3390/s100908363

**Published:** 2010-09-03

**Authors:** Ömer Galip Saracoglu, Hayriye Altural

**Affiliations:** Department of Electrical and Electronic Engineering, Erciyes University, 38039, Kayseri, Turkey; E-Mail: hayriye.altural@gmail.com

**Keywords:** color sensing, intelligent sensor, artificial neural network

## Abstract

A low-cost optical sensor based on reflective color sensing is presented. Artificial neural network models are used to improve the color regeneration from the sensor signals. Analog voltages of the sensor are successfully converted to RGB colors. The artificial intelligent models presented in this work enable color regeneration from analog outputs of the color sensor. Besides, inverse modeling supported by an intelligent technique enables the sensor probe for use of a colorimetric sensor that relates color changes to analog voltages.

## 1. Introduction

Color sensing is one of the important subjects of optical sensors. Color sensors have a variety of applications including detection of environmental, biological, and chemical parameters [1–7]. Color detection based chemical sensing is mostly implemented with particular emphasis on colorimetric sensors because many parameters, like pH [6], concentration [3], and chemical gases [4,8,9] can cause direct or indirect color changes in biological and chemical species.

Optical sensors usually have a non-linear relationship between the sensor’s response and the effect to be sensed or the measurand. Due to the fact that optical sensors have highly sensitive and non-linear nature, an unexpected change in the measurand may cause considerably changes and measurement errors in the sensor’s responses. Furthermore, it is expected that in modern sensor technology, a sensor has to adapt itself to the changing or unexpected conditions. In order to meet the expectations and to predict the sensor’s response more accurately, artificial intelligent techniques become a useful tool for the design of intelligent sensors [10–12].

Artificial Neural Networks (ANNs) are inspired by the brain’s complex, nonlinear, and parallel computing ability and therefore they have some exceptional properties for data processing, such as adaptation, learning, and generalization [13]. Intelligent optical sensors incorporate the abilities of ANNs with optical sensors inherently having high accuracy, long term stability, and immunity to electromagnetic interference. ANN based intelligent techniques have more significance if the sensor is highly nonlinear and/or a precise mathematical relationship cannot be established between the sensor’s response and the measurand. For example, when optical color sensors are used for classification of emerged colors, ANNs exhibit good performance in versatility of the measurement system [14,15].

The study we present in this paper is a low-cost reflective color sensor whose detection principle is similar to that of designs in [16–18] but overcomes the drawbacks reported therein with the aid of artificial intelligence. All parts of the sensor consist of cheap and easily available components. For example, the reflected signal from colored surface is produced by a RGB LED driven by a microcontroller and is detected by a photodiode. The analog signal from the photodiode is then amplified by an Op-Amp and applied to the microcontroller where it is converted to digital signals and processed. As it is well known, dark-colored surfaces degrade the performances of the reflective color sensors. In order to overcome this degradation and to improve sensor responses, we used ANN models utilizing multilayer perceptron (MLP) algorithms.

## 2. Brief Description of the Neural Networks

Neural Networks are perhaps the most popular intelligent technique in the design of intelligent sensors [19] and intelligent optical sensors [11,12]. This is especially because of their abilities in modeling of highly non-linear functions and generalizing of unseen data.

**Figure 1.**
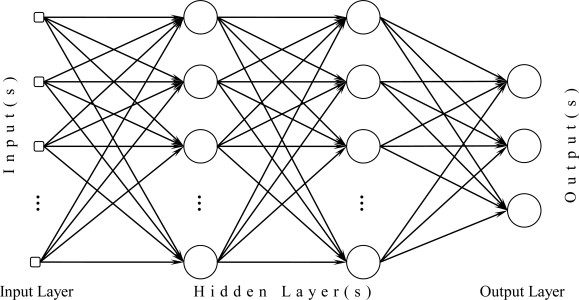
General structure of a multilayer perceptron (MLP).

There are different types of neural networks, such as multilayer perceptron (MLP), radial basis function (RBF) and generalized regression neural networks (GRNN), for modeling of non-linear functions and data estimation/prediction problems [20]. The three network types have not only many similarities but also significant discrepancies. Figure 1 [13] shows general structure of the three types of neural networks consisting of an input layer, one or more hidden layers and an output layer. The processing units of an MLP apply a linear function to their inputs while they typically have a non-linear activation function whereas the RBF and GRNN include radial processing units. Besides, MLPs can exhibit better performances when they are used for data estimation/prediction problems (see Table 3 of Foody *et al.* [20]) while some other network types such as RBF, GRNN and probabilistic neural network (PNN) can successfully solve classification problems [21]. Since our problem is typically a data estimation/prediction problem, we preferred to use MLP neural network whose brief description is given below. Performance comparisons of the network types may be a subject of further studies.

An MLP neural network consists of neurons (also called as nodes) connected to each other with weights. Each neuron has individual weights that are multiplied by the inputs when they enter to the processing unit. Then the processing unit sums up the inputs multiplied by the weights and produces the output of the neuron after the summation is applied to an activation function. The activation function must be a continuously differentiable function (preferably a non-linear function so that the network has a non-linear behavior). The function mostly used in MLP neural networks has sigmoidal nonlinearity whose types are logistic and hyperbolic tangent functions [13].

The input variables are transferred via the neurons at the input of the network. This group of the neurons is called input layer while the neurons producing of the network responses at the output are called output layer. An MLP network also includes one or more hidden layers between the two layers. In most nonlinear problems, two or more hidden layers improve the generalization ability of the network [13].

In order to train an MLP neural network, a learning algorithm is used to adjust the weights of the connections between the neurons in the layers. The performance of a learning algorithm generally changes from one problem to another and consequently, a trial-and-error method is mostly considered to determine the more efficient algorithm for a given problem. The learning algorithms having satisfactory performances used in this work are Gradient descent with momentum and adaptive learning rate backpropagation (GDX), Bayesian regularization backpropagation (BR), Levenberg-Marquardt backpropagation (LM), Resilient backpropagation (RP), and Broyden Fletcher Goldfarb Shanno quasi-Newton backpropagation (BFG).

GDX has an algorithm that updates weights and bias values according to gradient descent momentum and an adaptive learning rate. LM algorithm is a network training function that updates weight and bias values according to Levenberg-Marquardt optimization and it is the fastest (at the expense of the more memory usage) backpropagation algorithm in MATLAB Neural Network Toolbox. In BR algorithm, the weight and bias values are updated according to LM optimization that minimizes a combination of squared errors and weights. RP algorithm updates weight and bias values according to the resilient backpropagation algorithm and it works by modifying each weight by a learning parameter in order to minimize the overall error. Weights and bias values are updated according to the BFGS quasi-Newton method where the new weight is computed as a function of the gradient and the current weight. More details about the ANNs and learning algorithms can be found in [13,22].

## 3. Reflective Color Sensing

**Figure 2.**
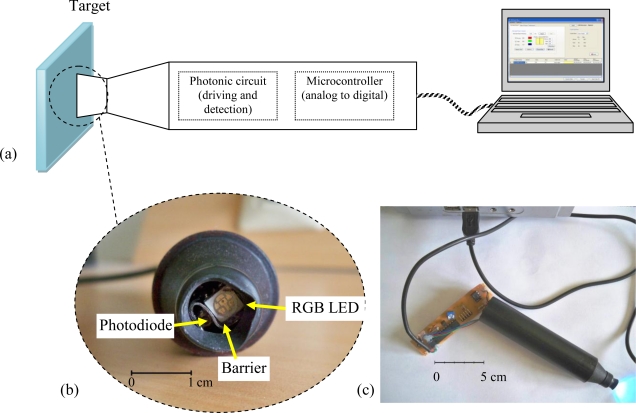
**(a)** Schematic illustration of RGB LED based color. **(b)** Photo of sensor tip. **(c)** Sensor probe connected with PC.

A typical reflective color sensor consists of three parts: a *target*, a *source* illuminating the target and a *detector* capturing the reflected light from the target. The most important parts of the design are the source and the detector. Reflective color sensors use either a broadband white-light source and three photo-detectors mounted behind of individual color filters or three narrow-light source generating RGB colors and a photo-detector. The sensor described in this section is the second and its general structure is given in Figure 2.

In a reflective color sensor, three phenomena take place when the light beams impinge onto the target: reflection, absorption, and transmission. A portion of incident light is reflected while the remaining is transmitted after a partial absorption regarding to the properties of the target. Two types of the reflected signal, *i.e*., diffuse and specular reflections, contribute to detector’s response. The diffuse reflection carries the useful information about the surface color; however varying specular reflection and fluctuating transparency can degrade the performance of the sensor. Fortunately, the specular reflection and the transparency remain almost constant for a given target and some small fluctuations in these parameters can be tolerated by intelligent approaches.

**Figure 3.**
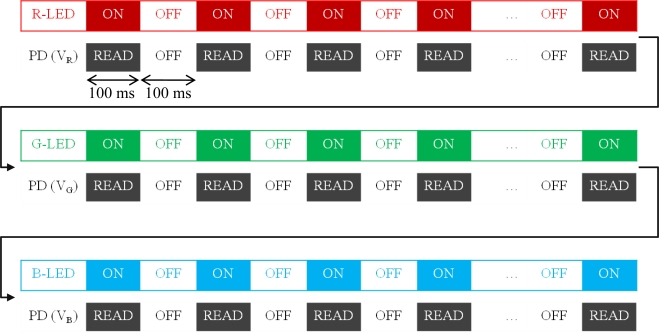
Switching of LEDs and the photodiode.

The cycle starts checking out whether the device is connected with PC. Then, minimum and maximum analog voltages produced by the probe are determined to calibrate the probe according to the properties of the surface to be measured. Minimum and maximum voltages are obtained from black and white colored surfaces, respectively. After calibration, the automatic measurement intervals are selected for each LED to be driven and the photodiode to be read. Time intervals are selected as 100 ms in this work. Microcontroller then drives R-LED during a duty-cycle of 100 ms as first and reads the photodiode’s output at the same time (Figure 3). This step is repeated averagely by 10 times until readouts remain stable. After completion of the R-LED period the microcontroller sends the readouts to the PC and initiates the G-LED period, and so on. When the three periods complete, (i) a new period can be started on the same surface, (ii) a new measurement can be started on another surface, or (iii) the procedure can be ended. A typical readout in terms of analog voltages and corresponding RGB contents is given in Table 1.

**Figure 4.**
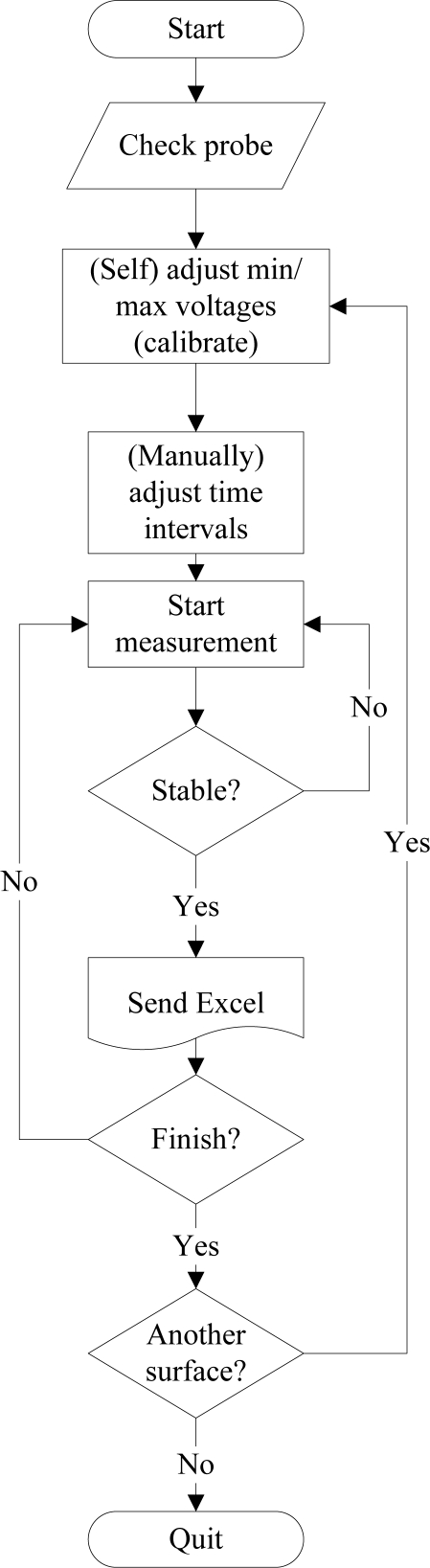
Flowchart of the measurement procedure.

The sensor uses RGB LEDs controlled by a microcontroller as the light source. A photodiode converts the optical signal reflected from the target into analog voltages. Then the microcontroller converts analog voltages into digital data and sends to computer after completion of a measurement cycle. This digital data is utilized to determine R, G, and B values, since the reflected intensity (*i.e.*, diffuse reflection) is proportional to RGB content of the target. Figure 4 describes how the measurement cycle completes.

## 4. Results and Discussion

An MLP neural network having an input layer with three inputs, two hidden layers, and an output layer with three outputs is used. The activation function of the output layer is a linear one while that of the other layers is the hyperbolic tangent function. The network is trained by five different training algorithms where the hidden neuron numbers in each layer are adjusted for the best performances. The inputs of the network are analog voltages obtained from the photonic circuit and the outputs are RGB contents of the surface. In order to obtain optimal network structures and neuron numbers in the hidden layers we used a trial and error approach. Although we started to train the networks with a minimum number of hidden layers and neurons in it, the best performances were obtained by different structures. Network structures of the proposed models are summarized in Table 2.

**Figure 5.**
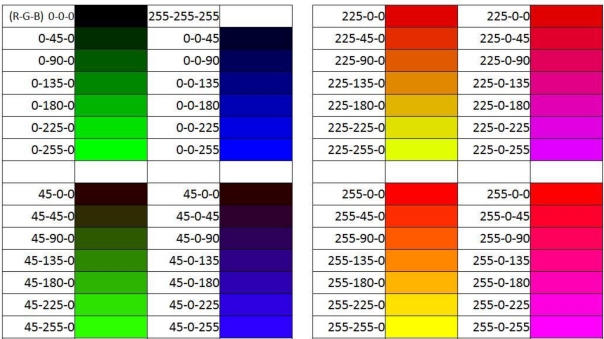
A part of color palette to constitute training/test dataset.

Although RGB content of any surface can be one of the 16 million possible combinations, we used a dataset consisting of only 246 data for training of the networks given in Table 2. Test dataset that is randomly selected and completely different from training dataset consists of 33 data. In order to constitute the dataset, we prepared a color palette whose a small part is given in Figure 5 and printed out them on a glossy paper by using a color laser printer. Then, for each color cell in the palette we obtained corresponding analog voltages by measuring with the probe given in Figure 2.

Computational performances of the networks are summarized in Table 3. It is useful to note that in the table, absolute error between the real and the networks’ values should be considered instead of usual error notations such as Root Mean Squared (RMS) and mean squared errors (MSEs), because absolute deviation from the real RGB content is more instructive than the others for this problem. Nevertheless, the MSEs of the models are also given in the table calculated with un-normalized values of the RGB contents. As can be seen from Table 3, BR and RP algorithms exhibit the best results in terms of maximum absolute error and time consumption.

For a better comparison of the performances, the best results (BR and RP) and the worst result (GDX) of the proposed models are given in Table 4. Numerical meaning of RGB contents is that if the absolute error is more than 30, human eye can distinguish the difference. In this context, RP and GDX results have two and four values, respectively, whose absolute errors are more than 30 while all of absolute errors of BR results are less than 30. Visual results of the networks are given in Table 5 as another performance comparison.

As can be seen from Table 5, all proposed networks can successfully model any RGB content of a given surface regarding analog voltages. In other words, the artificial intelligent models presented in this manner enable color regeneration from analog outputs of the color sensor (or from any voltage information). Besides, with the aim of an inverse modeling supported by an intelligent technique, the sensor probe can be used for a colorimetric sensor that relates color changes to analog voltages.

## 5. Conclusions

A low-cost optical sensor based on reflective color sensing is presented. Moreover, some neural network models are used as artificial intelligent technique to improve the color regeneration from the sensor signals. That is to say, analog voltages of the sensor can be successfully converted to RGB colors. The artificial intelligent models presented in this work enable color regeneration from analog outputs of the color sensor. Besides, an inverse modeling supported by an intelligent technique enables the sensor probe for use of a colorimetric sensor that relates color changes to analog voltages.

## Figures and Tables

**Table 1.** Typical photodetector readout in terms of analog voltages depending on RGB contents of the surface.

	**Photodetector readouts (volts)**			**RGB contents**		
Surface Color	V_R_, R-LED	V_G_, G-LED	V_B_, B-LED	R	G	B
Black	0.159	0.253	0.163	0	0	0
(Any)	0.800	1.25	1.77	135	90	225
White	3.67	3.66	3.66	255	255	255

**Table 2.** Network structures of the proposed models

**Training algorithm**	**Network type (neuron numbers in the layers)**			
	Input	1st hidden	2nd hidden	Output

Gradient descent with momentum and adaptive learning rate backpropagation (GDX)	3	8	9	3
Bayesian regularization backpropagation (BR)	3	10	5	3
Levenberg-Marquardt backpropagation (LM)	3	5	9	3
Resilient backpropagation (RP)	3	5	9	3
Broyden Fletcher Goldfarb Shanno quasi-Newton backpropagation (BFG)	3	12	9	3

**Table 3.** Network structures of the proposed models.

**Algorithm**	**Maximum absolute error**	**Un-normalized MSE**	**Epoch number**	**Time consumption (s)**
*GDX*	*38*	*268*	*75,000*	*416*
**BR**	**30**	**259**	**410**	**13**
LM	33	270	2,200	62
**RP**	**32**	**232**	**4,000**	**22**
BFG	34	265	2,200	110

**Table 4.** The best results (BR and RP) and the worst result (GDX) of the proposed networks.

No	Inputs (analog voltages)			Outputs (RGB contents)											
				Real values			BR results			RP results			GDX results			
	**V_R_**	**V_G_**	**V_B_**	**R**	**G**	**B**	**R**	**G**	**B**	**R**	**G**	**B**	**R**	**G**	**B**
1	0.88	1.59	0.48	**90**	**135**	**45**	**91**	**146**	**33**	**102**	**142**	**19**	**98**	**150**	**30**
2	2.14	1.62	0.46	**180**	**135**	**45**	**190**	**145**	**24**	**182**	**143**	**13**	**184**	**151**	**21**
3	0.35	0.73	0.91	**45**	**45**	**135**	**42**	**39**	**165**	**48**	**33**	**164**	**40**	**37**	**172**
4	1.00	0.57	0.69	**135**	**45**	**135**	**144**	**37**	**130**	**144**	**16**	**143**	**138**	**13**	**153**
5	3.55	0.71	0.87	**225**	**45**	**135**	**248**	**17**	**157**	**240**	**16**	**145**	**243**	**15**	**165**
6	1.30	0.93	0.38	**135**	**90**	**45**	**147**	**97**	**20**	**135**	**103**	**18**	**131**	**95**	**19**
7	1.91	3.64	0.73	**135**	**225**	**45**	**110**	**244**	**15**	**104**	**244**	**20**	**97**	**249**	**11**
8	0.54	1.83	1.46	**45**	**135**	**135**	**18**	**143**	**137**	**22**	**144**	**134**	**33**	**140**	**132**
9	0.94	3.25	1.27	**45**	**225**	**135**	**54**	**225**	**152**	**30**	**226**	**137**	**66**	**222**	**145**
10	0.58	0.64	0.93	**135**	**45**	**180**	**126**	**25**	**200**	**133**	**17**	**200**	**123**	**18**	**205**
11	0.60	1.77	0.82	**45**	**135**	**90**	**29**	**142**	**78**	**31**	**147**	**81**	**46**	**140**	**87**
12	0.48	1.96	2.58	**45**	**135**	**225**	**20**	**146**	**251**	**29**	**135**	**240**	**21**	**143**	**233**
13	1.36	1.93	0.90	**135**	**135**	**90**	**123**	**136**	**95**	**137**	**150**	**75**	**130**	**145**	**93**
14	3.66	1.89	0.97	**225**	**135**	**90**	**251**	**134**	**84**	**242**	**141**	**96**	**255**	**130**	**92**
15	1.28	1.06	1.11	**135**	**90**	**135**	**145**	**93**	**149**	**147**	**93**	**157**	**137**	**87**	**139**
16	3.66	1.00	1.01	**225**	**90**	**135**	**253**	**67**	**139**	**252**	**68**	**148**	**253**	**60**	**133**
17	1.80	3.00	1.01	**135**	**180**	**90**	**154**	**170**	**115**	**149**	**205**	**104**	**135**	**196**	**95**
18	0.88	2.03	1.60	**90**	**135**	**135**	**88**	**147**	**147**	**100**	**149**	**129**	**107**	**148**	**144**
19	1.23	3.64	1.48	**90**	**225**	**135**	**76**	**253**	**153**	**92**	**249**	**154**	**91**	**253**	**151**
20	0.80	1.25	1.77	**135**	**90**	**225**	**134**	**97**	**227**	**139**	**90**	**239**	**147**	**89**	**238**
21	0.63	1.97	2.59	**90**	**135**	**225**	**98**	**142**	**251**	**89**	**137**	**242**	**93**	**141**	**252**
22	2.36	1.73	1.19	**180**	**135**	**135**	**182**	**125**	**131**	**196**	**140**	**121**	**177**	**141**	**127**
23	1.61	2.96	1.55	**135**	**180**	**135**	**141**	**169**	**164**	**137**	**198**	**139**	**131**	**187**	**150**
24	1.17	1.98	2.07	**135**	**135**	**180**	**137**	**134**	**186**	**133**	**143**	**181**	**146**	**152**	**174**
25	1.94	1.62	1.64	**180**	**135**	**180**	**179**	**120**	**180**	**182**	**131**	**179**	**167**	**138**	**162**
26	3.65	1.88	1.85	**225**	**135**	**180**	**251**	**128**	**178**	**254**	**132**	**188**	**254**	**139**	**190**
27	2.16	2.93	1.40	**180**	**180**	**135**	**170**	**157**	**154**	**174**	**197**	**127**	**150**	**184**	**139**
28	1.45	3.01	2.36	**135**	**180**	**180**	**143**	**183**	**204**	**133**	**200**	**182**	**140**	**193**	**200**
29	1.53	3.65	2.17	**135**	**225**	**180**	**123**	**254**	**197**	**123**	**249**	**186**	**118**	**249**	**184**
30	3.09	3.66	2.06	**180**	**225**	**135**	**199**	**250**	**152**	**205**	**241**	**144**	**200**	**251**	**145**
31	1.65	1.75	2.16	**180**	**135**	**225**	**183**	**123**	**227**	**175**	**132**	**227**	**180**	**140**	**213**
32	2.95	1.74	2.11	**225**	**135**	**225**	**226**	**123**	**242**	**243**	**129**	**225**	**232**	**139**	**229**
33	1.67	3.65	3.56	**135**	**225**	**225**	**162**	**238**	**254**	**143**	**247**	**255**	**154**	**236**	**253**

**Table 5.** Visual comparisons of the real RGB contents and proposed neural network results.

No	Real	BR	RP	LM	BFG	GDX
	
1						
	
2						
	
3						
	
4						
	
5						
	
6						
	
7						
	
8						
	
9						
	
10						
	
11						
	
12						
	
13						
	
14						
	
15						
	
16						
	
17						
	
18						
	
19						
	
20						
	
21						
	
22						
	
23						
	
24						
	
25						
	
26						
	
27						
	
28						
	
29						
	
30						
	
31						
	
32						
	
33						
